# Predicting Phase 1 Lymphoma Clinical Trial Durations Using Machine Learning: An In-Depth Analysis and Broad Application Insights

**DOI:** 10.3390/clinpract14010007

**Published:** 2023-12-29

**Authors:** Bowen Long, Shao-Wen Lai, Jiawen Wu, Srikar Bellur

**Affiliations:** 1 Department of Analytics, Harrisburg University of Science and Technology, Harrisburg, PA 17101, USAsbellur@harrisburgu.edu (S.B.); 2 Zippin, Mill Valley, CA 94941, USA; judichunter@gmail.com

**Keywords:** trial duration, machine learning prediction, clinical research planning, lymphoma clinical trials

## Abstract

Lymphoma diagnoses in the US are substantial, with an estimated 89,380 new cases in 2023, necessitating innovative treatment approaches. Phase 1 clinical trials play a pivotal role in this context. We developed a binary predictive model to assess trial adherence to expected average durations, analyzing 1089 completed Phase 1 lymphoma trials from clinicaltrials.gov. Using machine learning, the Random Forest model demonstrated high efficacy with an accuracy of 0.7248 and an ROC-AUC of 0.7677 for lymphoma trials. The difference in the accuracy level of the Random Forest is statistically significant compared to the other alternative models, as determined by a 95% confidence interval on the testing set. Importantly, this model maintained an ROC-AUC of 0.7701 when applied to lung cancer trials, showcasing its versatility. A key insight is the correlation between higher predicted probabilities and extended trial durations, offering nuanced insights beyond binary predictions. Our research contributes to enhanced clinical research planning and potential improvements in patient outcomes in oncology.

## 1. Introduction

In the US, lymphoma has a significant impact on public health. It is estimated that 89,380 new cases will be diagnosed in 2023, ranking it among the top ten most frequently diagnosed cancers worldwide [1]. Tragically, the 5-year mortality rate for lymphoma exceeds 25% [1], and the disease is anticipated to claim over 21,080 lives within 2023 [1], highlighting the urgent need for innovative treatments. Phase 1 clinical trials play a crucial role in this arena, representing the first phase of human testing for investigational agents and turning years of lab research into actionable clinical solutions [2]. These trials form the foundation for later stages that emphasize efficacy and wider patient benefits. Their significance is clear: without these initial insights, the progression of novel treatments would be halted.

In this study, we aim to develop a binary predictive model to determine if trials will align with the average duration derived from our dataset, sourced from clinicaltrials.gov. Recognizing this benchmark is pivotal for several reasons:

- **Resource and Strategic Planning:** Predicting trial durations helps ensure optimal distribution of personnel and funds, minimizing inefficiencies. Furthermore, this foresight enables organizations to make informed decisions about trial prioritization, resource allocation, and initiation timelines [3,4];
- **Patient Involvement and Safety:** estimating trial durations provides patients with clarity on their commitment, which safeguards their well-being and promotes informed participation [5];
- **Transparent Relations with Regulators:** Providing predictions on trial durations, whether below or above the average, fosters open communication with regulatory authorities. This strengthens compliance, builds trust, and establishes transparent relationships among all stakeholders [6].

## 2. Background

As lymphoma diagnoses increase, precision in predicting Phase 1 lymphoma clinical trial durations has become crucial. Accurate predictions allow for efficient resource distribution, strategic foresight, enhanced patient participation and safety, and an open dialogue with regulatory authorities. Various studies, including research from *Nature*, have demonstrated that a variety of factors, such as strategic challenges, commercial barriers, operational setbacks, and unmanageable toxicity, frequently lead to unforeseen delays in clinical trials [7,8,9]. Multiple industry studies further emphasize this, noting that nearly 85% of trials experience setbacks [10], highlighting the pressing need for reliable duration prediction tools or calculators.

In the realm of clinical trial calculators, the predominant focus lies on calculating sample size (the number of subjects needed for adequate statistical power) [11,12,13]. However, there is a notable absence of widely used calculators or applications that provide estimated clinical trial durations based on enrollment numbers and various trial design variables. Given the scarcity of research on trial duration estimation, the existing studies often focus solely on adverse events as an outcome measure to determine duration. This approach overlooks trials with multiple outcome measures and neglects various non-outcome measure variables, such as enrollment, trial locations, intervention types, sponsors, and study patterns, all of which collectively influence estimation. For example, LV Rubinstein et al. [14] used a parametric test based on exponential death time assumptions to provide trial length calculations for statistical power. However, its heavy reliance on parametric assumptions and limited consideration of non-exponential death times or other outcome measures restrict its broad applicability. Furthermore, existing research frequently neglects the integration of large, actual historical trial datasets to derive duration insights. Camerlingo, Nunzio et al. [15] developed a formula to determine the minimum trial duration needed to evaluate glucose control time-in-ranges for desired values. However, for practical use, it requires extensive real-world experimentation in clinical trials due to its reliance on simulated data from continuous glucose monitoring (GCM). In contrast, our research introduces a machine learning-based clinical trial duration calculator capable of handling a diverse array of trial-specific variables and integrating large volumes of actual historical clinical trials, thereby providing a more robust and comprehensive duration estimation.

Machine learning has shown immense promise in clinical trials for aspects like trial design, patient recruitment, outcome predictions, and regulatory adherence. A deeper dive into the existing literature, however, reveals a distinct lack of research on using machine learning to predict clinical trial durations, especially regarding Phase 1 lymphoma trials. There are noteworthy machine learning applications in various trial phases, such as using machine learning to optimize trial design for ARDS patients in ICUs [16], forecasting early trial terminations [17], and refining trial design to improve success rates [18]. In patient recruitment, machine learning combined with EHR data and NLP has been employed for patient eligibility [19] and participant identification [20]. A wealth of studies also exist on outcome predictions using machine learning, from predicting treatment responses [21] to forecasting outcomes based on patient profiles [22] to predicting negative outcomes, with an emphasis on mortality events [23]. In regulatory compliance, machine learning has been used to automate clinical research classification [24] and recommend regulatory strategies [6].

However, in this vast landscape, the specific application of machine learning for clinical trial duration prediction remains largely untapped. One pertinent study used a gradient-boosted tree-based model on Roche’s dataset to gauge trial efficiency, albeit not concentrating on duration prediction [4]. Currently, a significant gap exists in applying machine learning models for clinical trial duration predictions—a void our research intends to fill. We are at the forefront of this domain, showcasing how machine learning can predict clinical trial timeframes. Our study not only addresses a significant gap in the literature but also stresses the importance of duration prediction in clinical trial planning and resource allocation. Given the unpredictable nature of continuous outcomes in clinical research [25,26,27], our technique leans towards binary prediction. Instead of estimating exact durations, our model evaluates whether a trial will be shorter or longer than the average duration derived from the clinicaltrials.gov dataset. This approach aligns with recent trends in oncology predictions [28,29,30], presenting several benefits. Notably, the binary framework is less influenced by outliers, reducing distortions from extreme values [31,32,33,34]. By categorizing results into distinct, actionable groups, our model brings clarity and ensures a balance between practicality and prediction reliability.

Key Contributions:

- **Pioneering Work in Duration Prediction:** our machine learning model stands as a trailblazing effort in the domain, bridging the existing gap in duration prediction applications and establishing benchmarks for future research;
- **Diverse Modeling:** we extensively reviewed eight machine learning models, highlighting the Random Forest model for its unparalleled efficiency in predicting durations;
- **Comprehensive Variable Exploration:** our model incorporates varied variables, from enrollment metrics to study patterns, enhancing its predictive capabilities;
- **Insight into Data Volume:** beyond mere predictions, we delve into determining the optimal data volume required for precise forecasting;
- **In-Depth Model Probability:** Apart from binary predictions, our model associates higher probabilities with longer average durations, along with a 95% CI. This precision offers a comprehensive range of potential trial durations, aiding informed decision-making and strategic planning;
- **Broad Applicability:** with proven efficacy in lung cancer trials, our model showcases its potential use across various oncology areas.

## 3. Materials and Methods

### 3.1. Dataset

We sourced our dataset from clinicaltrials.gov [35], a prominent global registry for clinical research studies. Our research focused exclusively on Phase 1 trials related to ‘Lymphoma’ that had started before 2023 and were marked as ‘Completed’. This approach resulted in a collection of 1231 studies. The decision to exclude trials conducted in 2023 was primarily driven by concerns related to seasonality. Given that we are only halfway through the year, data from 2023 may not provide a comprehensive understanding of the seasonal factors affecting trial durations.

For external validation, we gathered data on 907 completed Phase 1 trials related to ‘Lung Cancer’ up to the same reference date. This decision was guided by several considerations. First, clinical trial data for lung cancer studies were sufficiently comprehensive on clinicaltrials.gov, enabling us to compile a dataset with a comparable level of detail to our primary lymphoma dataset. Second, lung cancer, as a well-studied and prevalent cancer type, adds a layer of diversity to the external validation process, enhancing the robustness and generalizability of our predictive model across different oncological contexts. Last, the commonalities in trial design, regulatory requirements, and clinical trial endpoints between lymphoma and lung cancer trials [36,37] make the external validation a relevant and meaningful test of our model’s performance.

Table 1 provides an overview of the dataset’s columns using an example trial. The ‘Duration’ variable was computed by calculating the time interval between the ‘Start Date’ and the ‘Completion Date’. The average duration of Phase 1 lymphoma trials was found to be 1788 days, roughly equivalent to 5 years. Subsequently, we established a binary prediction target based on this 5-year benchmark. The remaining variables in our dataset were utilized as predictors for our model. In the dataset, approximately 40% of trials exceeded this benchmark, while around 60% fell below it.

### 3.2. Data Preprocessing

To build an appropriate predictive model for Phase 1 lymphoma clinical trial durations, we conducted data preprocessing. We first removed trials with missing start or completion dates, reducing the lymphoma dataset from 1231 to 1089 studies. We split this data into 80% for training and 20% for testing, and we used 5-fold cross-validation for hyperparameter tuning and model selection. We addressed missing values by imputing the mean for numerical variables like enrollment in the lymphoma data. Categorical variables with missing values were treated as a separate category. For the lung cancer dataset, which served as external validation, we followed a similar process, reducing the dataset from 907 to 840 studies. We imputed missing values in the enrollment variable with the mean and treated missing values in categorical variables as a separate category.

### 3.3. Data Exploration and Feature Engineering

**Figure 1:**
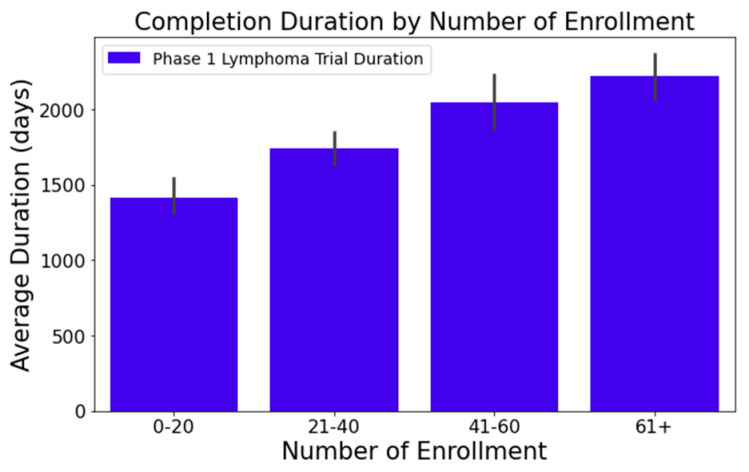
Impact of enrollment numbers on trial duration.

**Figure 2:**
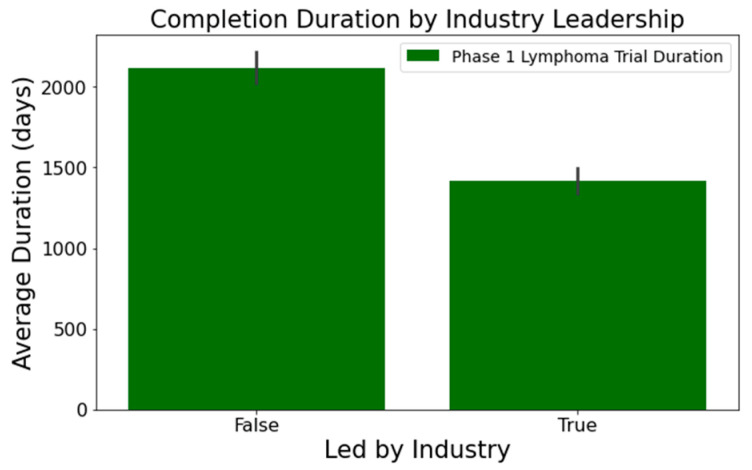
Impact of industry leadership on trial duration.

**Figure 3:**
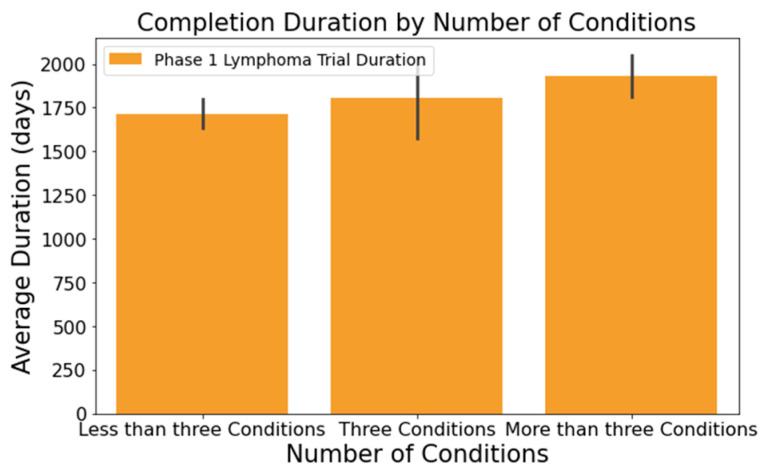
Impact of condition count on trial duration.

**Figure 4:**
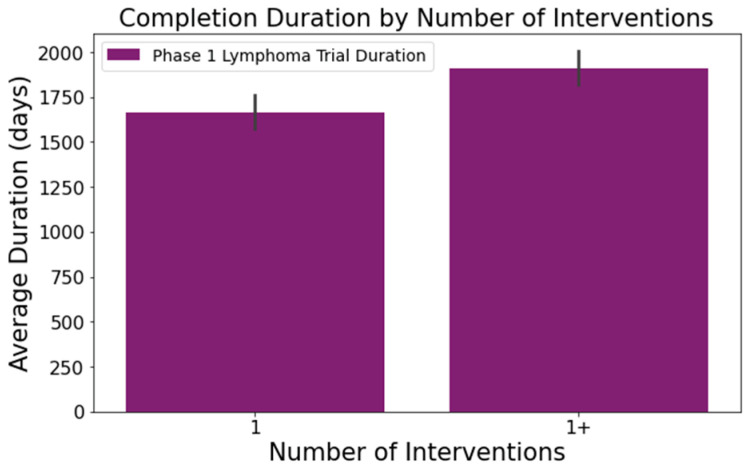
Impact of intervention count on trial duration.

**Figure 5:**
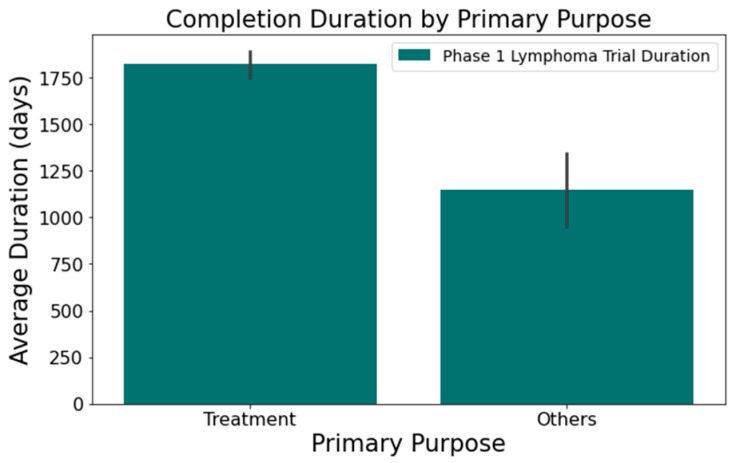
Impact of trial focus on trial duration.

Upon analyzing the lymphoma clinical trials dataset, we pinpointed several columns significantly influencing the clinical trial duration. These include the following:

- Figure 1 illustrates that trials tend to take longer with increased enrollment. For example, trials with 0–20 enrollees averaged about 1417 days, while those with 61 or more enrollees extended to 2218 days—roughly 1.6 times longer. This difference was statistically significant at a 95% confidence level;
- In Figure 2, industry-led trials concluded more quickly than non-industry-led ones. On average, industry-led trials (510 trials) had a mean duration of 1414 days, notably shorter than non-industry-led trials with a mean duration of 2118 days across 579 trials. This difference was statistically significant at a 95% confidence level;
- The number of conditions or interventions in a trial correlates with its duration, as indicated in Figure 3 and Figure 4. For instance, trials with fewer than three conditions lasted about 1714 days (Figure 3). Those with more than three conditions had a 215-day longer mean duration (1929 days), with statistical significance across the three groups at a 95% confidence level. In Figure 4, trials with more than one intervention, on average, took 248 days longer to complete than those with only one intervention (1661 days), a statistically significant difference at 95%;
- Figure 5 shows that the primary purpose significantly affects the trial duration. ‘Treatment’ trials had a mean duration of 1821 days, while trials with other primary purposes were completed almost two years quicker, with a mean duration of 1145 days. This difference was statistically significant at a 95% confidence level.

**Figure 6:**
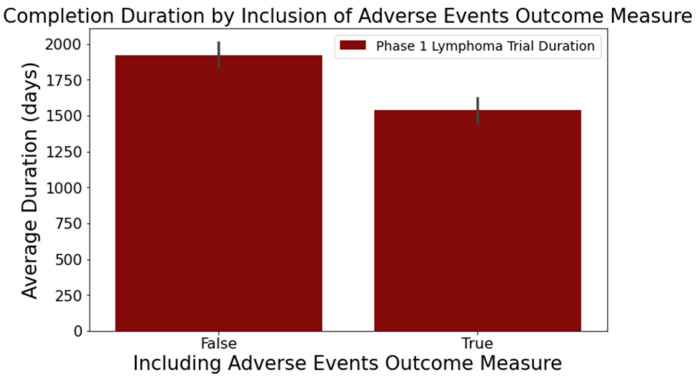
Impact of adverse event outcome measure on trial duration.

**Figure 7:**
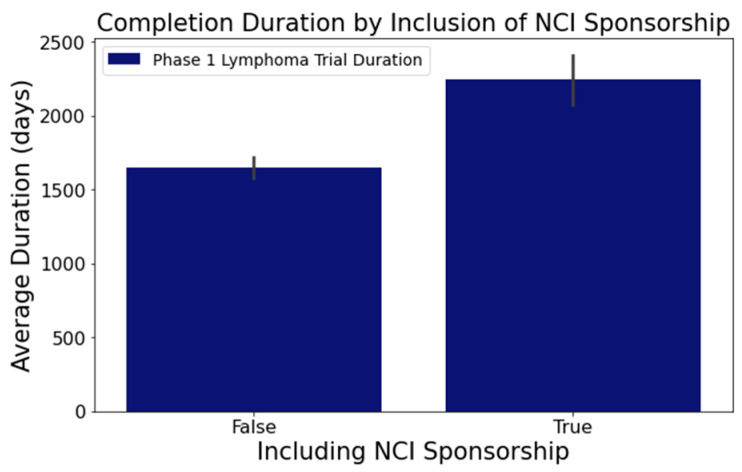
Impact of NCI sponsorship on trial duration.

**Figure 8:**
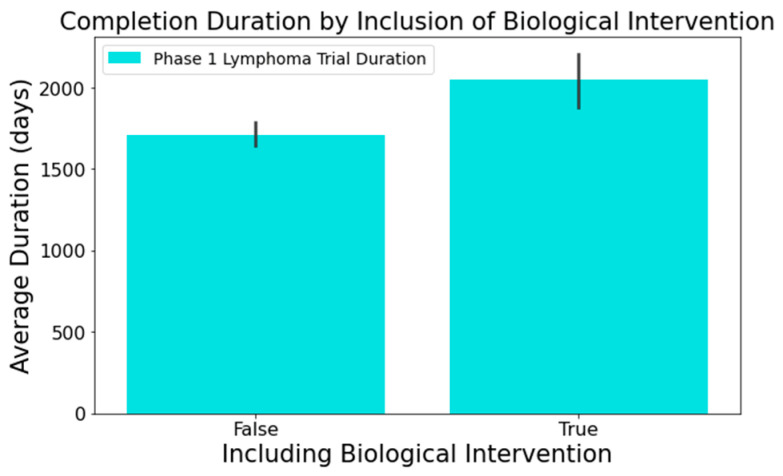
Impact of biological intervention on trial duration.

In columns with substantial textual data, such as ‘Outcome Measures’ and ‘Sponsor/Collaborators’, we employed the spaCy library [38] to determine semantic resemblance between terms. Words with a similarity score surpassing 0.8 were grouped using a Disjoint Set Union (DSU) approach [39], enhancing the categorization beyond mere string matching. For example, terms such as ‘adverse events’, ‘adverse reactions’, and ’aes’ all relate to the ‘Outcome Measures’ category for adverse events. Notable findings from this analysis segment include the following:

- Figure 6 reveals that trials focusing on adverse events in the ‘Outcome Measures’ column tend to conclude faster. Trials without adverse event measurement had a mean duration of 1919 days across 716 trials, while those with such measurement had a mean duration of 1537 days across 373 trials. This difference was statistically significant at a 95% confidence level;
- In Figure 7, trials indicating the ‘National Cancer Institute (NCI)’ as a sponsor tended to have longer durations. Trials with NCI sponsorship had a mean duration of 2246 days, compared to trials without, which had a mean duration of 1648 days. This difference was statistically significant at a 95% confidence level;
- Figure 8 highlights that the involvement of biological interventions in trials often results in extended durations, a statistically significant difference at 95%.

These insights from our exploratory data analysis informed our feature creation for modeling. Following iterative selection, we incorporated 30 features into our models. Table 2 below enumerates these features, ranked in descending order of importance, as determined by Gini Gain [40].

### 3.4. Machine Learning Models and Evaluation Metrics

Using Python 3.9.7, we selected eight distinct machine learning models/classifiers to predict the duration of lymphoma clinical trials. Our choices were informed using previous research in oncology clinical trial predictions [4,6,16,17,18,19,20,21,22,23,24] and the inherent strengths of each model. These models are Logistic Regression (LR), K-Nearest Neighbor (KNN), Decision Tree (DT), Random Forest (RF), XGBoost (XGB), Linear Discriminative Analysis (LDA), Gaussian Naïve Bayes (Gaussian NB), and Multi-Layer Perceptron Classifier (MLP).

Each model underwent a thorough evaluation of the lymphoma dataset. To refine the models and achieve optimal results, we used the GridSearchCV (GSCV) technique from the Scikit-Learn library [41]. GSCV effectively helps in hyperparameter tuning by cross-validating the classifier’s predictions and pinpointing the best parameter combination for peak performance.

#### 3.4.1. Logistic Regression (LR)

We started with Logistic Regression for its simplicity and clarity. We utilized the LogisticRegression function from Scikit-Learn’s linear_model library [41]. However, its linear decision boundary might fall short of capturing complex data relationships.

#### 3.4.2. K-Nearest Neighbors (KNN)

To address the limitations of linearity, we next looked to KNN, an instance-based learning method that classifies based on data similarity. We implemented KNN using the KNeighborsClassifier from Scikit-Learn’s neighbors library [41]. Given its computational intensity, especially with a relatively higher number of features, we sought more computationally efficient models, leading us to tree-based options, starting with the Decision Tree (DT).

#### 3.4.3. Decision Tree (DT)

Decision Trees offer a more expressive way of modeling. We implemented the model using the DecisionTreeClassifier function from Scikit-Learn’s tree library [41]. However, their susceptibility to overfitting led us to consider ensemble techniques such as Random Forest and XGBoost.

#### 3.4.4. Random Forest (RF) and XGBoost (XGB)

Random Forests and XGBoost leverage the collective strength of multiple trees. Specifically, Random Forest aggregates trees using bagging, while XGBoost refines predictions sequentially through a boosting mechanism. We implemented Random Forest using the RandomForestClassifier from Scikit-Learn’s ensemble library [41] and XGBoost using the XGBClassifier function from the XGBoost library [42].

#### 3.4.5. Linear Discriminant Analysis (LDA) and Gaussian Naïve Bayes (Gaussian NB)

Transitioning from discriminative models like Logistic Regression, K-Nearest Neighbors, and tree-based methods, we integrated Linear Discriminant Analysis (LDA) and Gaussian Naïve Bayes (Gaussian NB) to explore a probabilistic approach.

LDA seeks to maximize class separation by identifying the linear combination of features that best distinguish between classes. This method presupposes that features within each class are normally distributed with identical covariance matrices. On the other hand, Gaussian NB is grounded in Bayes’ theorem, operating under the assumption of feature independence.

We employed the LinearDiscriminantAnalysis function for LDA and the GaussianNB function for Gaussian NB, both sourced from Scikit-Learn [41]. Recognizing the stringent assumptions of these methods, we turned our attention to models renowned for their flexibility and potential for high accuracy, specifically neural networks.

#### 3.4.6. Multi-Layer Perceptron (MLP)

Concluding our model selection, we turned to the Multi-Layer Perceptron, a neural network renowned for its ability to model complex relationships without being bound by strict data assumptions. However, MLP’s “black box” nature makes it less transparent compared to models like Logistic Regression and Decision Trees. This can hinder its interpretability in critical scenarios. We implemented MLP using Scikit-Learn’s MLPClassifier from the neural_network library [41].

To assess the effectiveness of our classifiers, we employed established metrics, specifically accuracy, Area Under the Curve (AUC) of the Receiver Operating Characteristic (ROC), precision, recall, and F1-score. These metrics are grounded in the values of true positives (TP), false positives (FP), false negatives (FN), and true negatives (TN):

- Accuracy measures the fraction of correct predictions (see Equation (1));
- ROC visually represents classifier performance by plotting recall against the false positive rate (see Equation (2)) across diverse thresholds. This visual representation is condensed into a metric via the AUC, a value between 0 and 1, where 1 signifies flawless classification;
- Precision gauges the reliability of positive classifications, shedding light on the inverse of the false positive rate (see Equation (3));
- Recall (or sensitivity) denotes the fraction of actual positives correctly identified, emphasizing the influence of false negatives (see Equation (4));
- F1-score provides a balance between precision and recall, acting as their harmonic mean (see Equation (5)).

(1) $$Accuracy=\frac{TP+TN}{TP+FP+FN+TN}$$

(2) $$False\;Positive\;Rate=1-\frac{TN}{TN+FP}=\frac{FP}{FP+TN}$$

(3) $$Precision=\frac{TP}{TP+FP}$$

(4) $$Recall=\frac{TP}{TP+FN}$$

(5) $$F1\;Score=\frac{2\times Precision\times Recall}{Precision+Recall}=\frac{2TP}{2TP+FP+FN}$$

## 4. Results and Discussion

### 4.1. Sample Characteristics

In our model for predicting the duration of Phase I clinical trials for lymphoma, we partitioned our data such that 80% was used for training and validation, employing a 5-fold cross-validation technique. The remaining 20% was reserved for testing. Table 3 provides a detailed breakdown of the main attributes of our datasets, spotlighting the 12 most salient features identified in Section 3—Data Exploration, for both the training/cross-validation and testing sets.

The table illustrates the similarities between our training/cross-validation and testing datasets across various attributes. Notably, both sets have an equivalent distribution of the target variable, with 40% of trials taking over 5 years to complete. Metrics like Average Enrollment of Trial Participants and Percentage of Trials Led by Industry show only slight variations. This uniformity across key characteristics supports the appropriateness of the data split for model training and testing.

### 4.2. Machine Learning Classification

Table 4 assesses the prediction capabilities of eight machine learning classifiers using a 5-fold cross-validation approach. Results were presented as average values within a standard deviation. While all these metrics hold significance in gauging a model’s forecasting ability, we primarily focused on accuracy, followed by the ROC-AUC metric.

Both XGBoost (XGB) and Random Forest (RF) demonstrated strong performance metrics. XGBoost achieved an accuracy of 74.42%, an ROC-AUC score of 78.54%, and a precision of 70.09%, and Random Forest followed closely with an accuracy of 73.71% and an ROC-AUC of 77.55%, emphasizing its notable predictive prowess. Although their average metrics were similar, Random Forest exhibited more variability in parameters like recall (62.86 ± 9.69%). Logistic Regression (LR) and Linear Discriminant Analysis (LDA) provided comparable results, with accuracies of 71.18% and 70.72% and respective ROC-AUC scores of 77.60% and 75.67%. The Multi-Layer Perceptron (MLP) registered an accuracy of 67.17% and an ROC-AUC of 70.71%. However, its recall’s higher standard deviation (49.14 ± 9.84%) hinted at potential inconsistencies across runs. Gaussian Naïve Bayes notably achieved a high recall of 90.86%, but its accuracy is compromised, given its score of 52.93%. K-Nearest Neighbors (KNN) and Decision Tree (DT) lagged in performance, indicating potential areas for improvement.

For the top four models with the highest accuracy and ROC-AUC scores (XGBoost, Random Forest, Logistic Regression, and Linear Discriminative Analysis), we further compared their performance on the testing set. As detailed in Table 5, Random Forest continues to be the top-performing model on the testing set, consistent with its strong performance in cross-validation. It exhibits the best accuracy, ROC-AUC, and F1-score. Linear Discriminative Analysis (LDA) has moved to the second position in terms of accuracy and F1-score, showing robust generalization, possibly due to its assumptions about the underlying distribution of the data that enable it to derive optimal decision boundaries between classes [33]. Interestingly, XGBoost, which was the top model in cross-validation, now has relatively lower accuracy than Random Forest and Linear Discriminative Analysis. This shift might be attributed to overfitting during cross-validation, where the model could have captured noise in the training data that did not generalize well. Logistic Regression maintains its position, showcasing consistent but comparatively lower performance.

**Figure 9:**
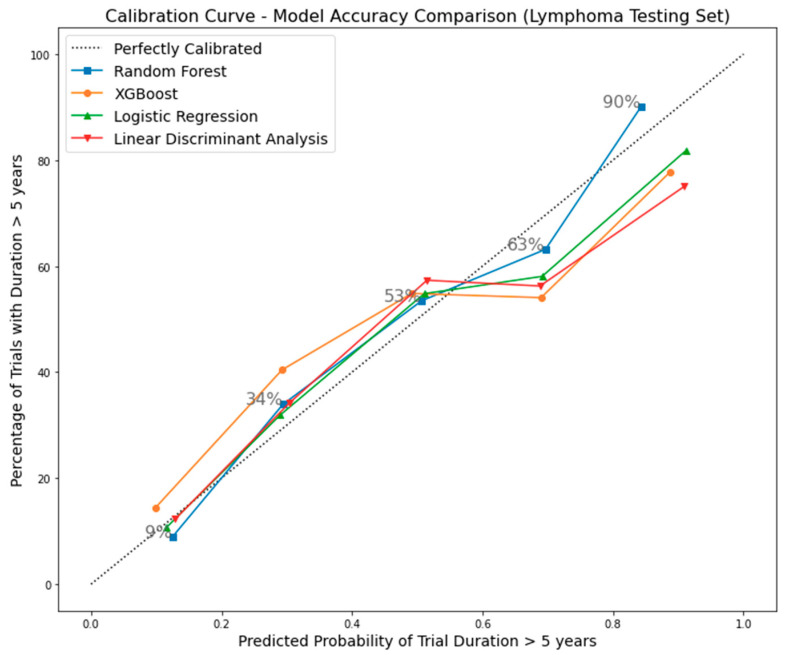
Calibration curve comparing Random Forest accuracy with other models in clinical trial duration prediction.

To intuitively assess how Random Forest’s accuracy compares with that of Linear Discriminative Analysis, Logistic Regression, and XGBoost models in clinical trial duration, we utilized the calibration curve in Figure 9. This curve depicts the percentage of actual trials lasting over 5 years against the predicted probability of exceeding this duration. All four top models are compared against the perfectly calibrated line (the diagonal). In the figure, within the predicted probability range of 0–0.6, all models closely align with the calibrated line, except for XGBoost. Beyond a predicted probability of 0.6, especially within the 0.6–0.8 range, all models deviate further. Notably, Random Forest remains the closest fit among the four. Beyond a predicted probability of 0.8, other models do not return to the proximity observed in the 0–0.6 range. In contrast, Random Forest strives to return from deviation and maintains optimal performance, similar to the 0–0.6 range. Furthermore, to determine whether there is a statistically significant difference in accuracy levels between Random Forest and the other three models, we conducted chi-square tests. These tests compared the number of correctly predicted trials using Random Forest with each of the other three models individually, as shown in Table 6. The results indicate that all *p*-values are less than 0.05, suggesting that the differences in accuracy levels between Random Forest and the other three models are statistically significant at a 95% confidence interval.

In conclusion, the Random Forest model consistently outperformed in accuracy, ROC-AUC, and other key metrics on both cross-validation and out-of-sample testing datasets, and the difference in accuracy levels is statistically significant at a 95% confidence interval. Thus, we advocate for its adoption as the most reliable model to predict the duration of phase I lymphoma clinical trials. Table 7 delves into the parameter tuning for this model. In our grid search for Random Forest, we experimented with tree counts ranging from 50 to 500; max depths of none, 10, 20, and 30; min sample splits of 2, 5, and 10; and explored both bootstrap options. The best-performing configuration utilized a max depth of 20, a min samples split of 10, 100 trees, and no bootstrap.

**Figure 10:**
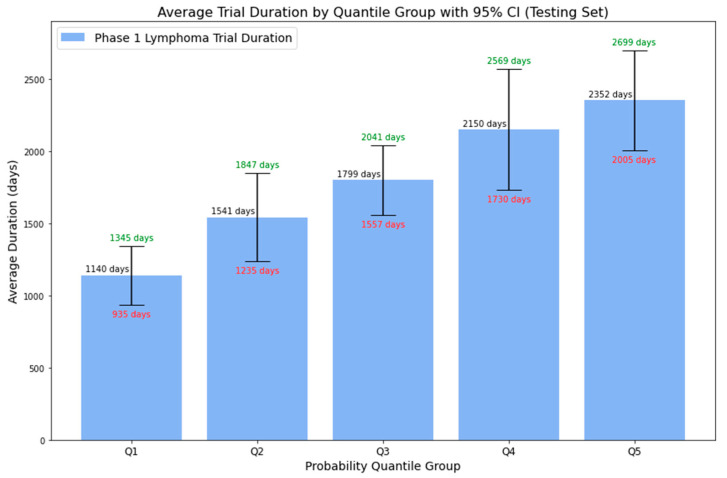
Average duration of Phase 1 lymphoma trials by probability quantile group.

With the final trained Random Forest model, we forecasted the probability of a Phase 1 trial exceeding a duration of five years on the lymphoma testing set. Figure 10 displays the average duration of Phase 1 lymphoma trials across 5-quantile probability groups, with associated 95% confidence intervals. The data show an increasing trend: the average duration rises with higher predicted probabilities. Specifically, the average duration is around 3.12 years (or 1140 days) for the first quantile group and approximately 6.44 years (or 2352 days) for the fifth quantile group. Furthermore, for all probability groups, the upper bounds of the 95% CI correlate with higher predicted probabilities, while the lower bounds follow the inverse pattern. This enhanced representation provides more than just a binary outcome, offering stakeholders a detailed range of potential trial durations complete with confidence intervals. Such precision aids in better decision making and strategic planning, turning uncertainties into clear, actionable insights for efficient clinical trial management. Table 8 delineates the corresponding probability range by quantile groups based on the results from the lymphoma testing set.

### 4.3. Random Forest Model Validation

#### 4.3.1. Impact of Varying Training Data Sizes on Model Performance

**Figure 11:**
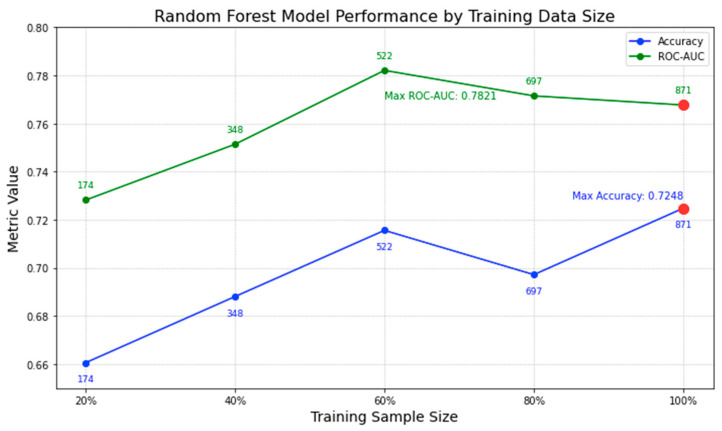
Random Forest model performance by training data size.

In Figure 11, we illustrate the performance trajectory of our random forest model on lymphoma testing data with increasing training sizes. An interesting trend emerges: while there is a positive correlation between training size and accuracy, the incremental gains in accuracy diminish as the dataset size increases. For instance, the leap in accuracy from 20% to 60% training size is notable, but after the 60% mark, the growth rate tapers. By the time we reach our full set of 871 trials (highlighted by the red dot), the model achieves an accuracy peak of 0.7248. It is noteworthy that even though the highest ROC-AUC is recorded at 60% data usage, the difference in comparison to the full dataset is slim. This subtle increase in accuracy, coupled with the broadened data spectrum when using all 871 trials, assures us of a well-generalized model. The current analysis underscores our confidence in the 871-trial dataset; additional data from clinicaltrials.gov might refine the model further, but the likelihood of a significant boost in efficiency is marginal.

#### 4.3.2. External Validation Using Phase 1 Lung Cancer Trial Data

**Figure 12:**
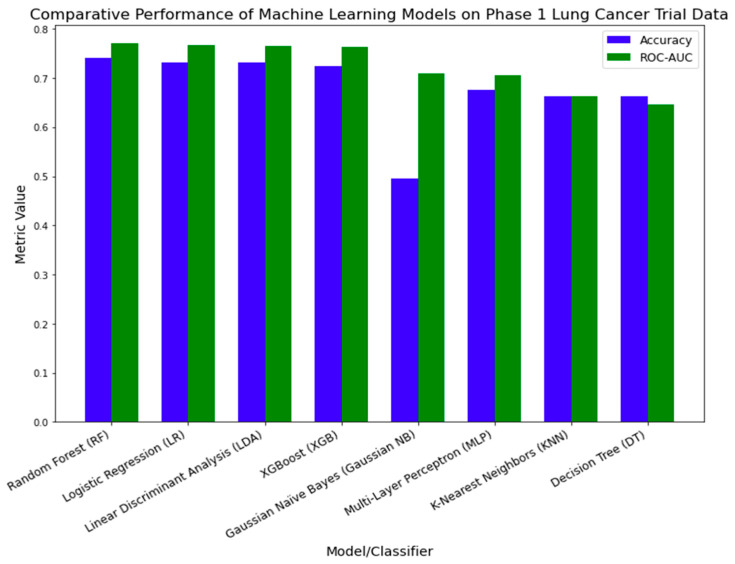
Comparative performance of machine learning models on Phase 1 lung cancer trial data.

In the external validation process, we evaluated the efficacy of eight machine learning models on Phase 1 lung cancer trial data. Figure 12 illustrates the performance metrics for each model. The Random Forest (RF) classifier demonstrated the highest performance, with an accuracy of 0.7405 and an ROC-AUC of 0.7701. Logistic Regression (LR) and Linear Discriminant Analysis (LDA) followed, registering accuracy rates of 0.7321 and 0.7310 and ROC-AUC values of 0.7671 and 0.7647, respectively. Despite its recognized robustness in a variety of healthcare applications [43,44,45,46], XGBoost (XGB) was ranked fourth, with an accuracy of 0.725 and an ROC-AUC of 0.7632. The other models displayed relatively lower performance metrics.

**Figure 13:**
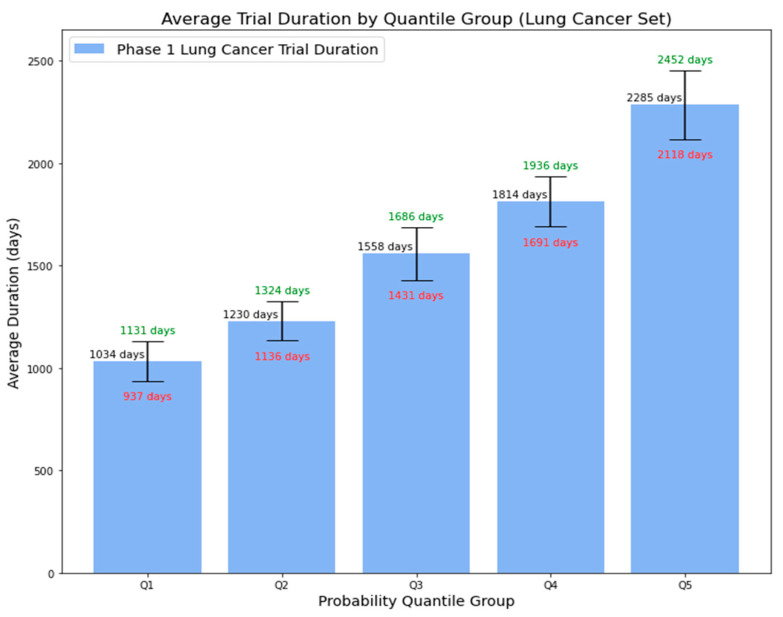
Average duration of Phase 1 lung cancer trials by probability quantile group.

Utilizing the final trained Random Forest model, similar to our approach with the lymphoma dataset, we predicted the probability of a Phase 1 lung cancer trial extending beyond five years. Figure 13 presents the average duration of Phase 1 lymphoma trials, grouped by 5-quantile probability, accompanied by 95% confidence intervals. There was a clear trend: trials with higher predicted probabilities tended to have longer average durations. This trend, observed in both lymphoma and lung cancer trials, not only supplements the simple binary output regarding whether the trial is likely to be below or beyond 5 years but also provides essential insights for stakeholders in planning and resource allocation for clinical trial research.

Considering the cross-validation and testing results from the lymphoma dataset, the consistent performance of the Random Forest model was evident. These outcomes further justify our selection of Random Forest as the optimal model for forecasting Phase 1 lymphoma clinical trials. The Random Forest classifier’s consistency across both datasets suggests its potential general applicability for Phase 1 clinical trial predictions.

##### Limitations

This study, while providing valuable insights, comes with certain limitations that merit consideration. Primarily, our dataset was exclusively extracted from clinicaltrials.gov, which, although a comprehensive platform, does not cover all Phase 1 lymphoma trials worldwide. This may introduce biases or omit nuances evident in trials recorded in other databases or those from different regions. Furthermore, the decision to eliminate trials with missing start or completion dates, while methodologically sound, could inadvertently exclude particular patterns or outliers that are relevant [47]. Employing mean imputation as a method to address missing values, while a common practice, has its limitations as it can reduce the variance and might influence the predictive power of our models [48]. The external validation with the lung cancer data strengthens our findings, but it also emphasizes the need for further validations across various cancer types to understand the comprehensive applicability of our model. Finally, while the Random Forest model demonstrated consistency across the datasets, the inherent variability and intricacies of clinical trials, even within the same phase or disease type, could impact its generalizability. Enhancing the model’s general applicability might be achieved by incorporating more diverse datasets, adding domain-specific features, or refining preprocessing strategies to account for these complexities.

## 5. Conclusions

In our analysis of Phase 1 lymphoma clinical trials from clinicaltrials.gov, we pinpointed 30 significant factors affecting trial durations. For instance, trials with larger enrollments usually had extended durations, while industry-led efforts concluded more promptly. Trials linked to the ‘National Cancer Institute (NCI)’ or those examining a more extensive range of conditions or interventions generally took longer. Conversely, trials concentrating on adverse event measurements ended more rapidly.

Of the eight machine learning models we evaluated, the Random Forest classifier stood out as the most effective. It achieved an accuracy of 0.7248 and an ROC-AUC score of 0.7677 on the lymphoma trial testing dataset. Adjusting the training data size revealed that accuracy gains began to level off after using 60% of the data. This indicates that our chosen dataset size is close to optimal for this analysis. Notably, when tested on Phase 1 lung cancer trial data, the classifier achieved an accuracy of 0.7405 and an ROC-AUC of 0.7701, underscoring its adaptability beyond just lymphoma trials. This points to its potential for predicting durations for a broader set of Phase 1 clinical trials.

Going deeper, we carried out a thorough analysis of average durations by predicted probability groups. This additional exploration provided stakeholders with more precise duration estimates, accompanied by a 95% confidence interval for each group. This information is invaluable for strategic planning, resource allocation, and risk mitigation.

By predicting Phase 1 lymphoma trial durations, distinguishing between longer and shorter trials, and providing a 95% confidence interval for the duration range, our model addresses critical aspects of clinical trial management, aids in strategic planning, and has the potential to enhance patient outcomes. Clinical trial delays can incur costs ranging from USD 600,000 to USD 8 million daily [49]. Our model plays a role in addressing these potential costs by contributing to efficient trial resource allocation and management, the saved cost of which could be redirected toward further drug development, ultimately enhancing patient outcomes. Moreover, considering the 10% failure rate in clinical drug development due to factors like poor strategic planning [8], our research plays a pivotal role in aiding resource management and strategic planning through duration predictions, potentially improving success rates. While acknowledging the complexities of drug development, our research offers a tangible step toward more efficient, cost-effective, and successful Phase 1 clinical trials, ultimately benefiting patients and advancing oncology research.

## Figures and Tables

**Table 1:** Overview of columns in the Phase 1 lymphoma trial dataset using an example trial.

Column	Value
NCT Number	NCT02220842
Title	A Safety and Pharmacology Study of Atezolizumab (MPDL3280A) Administered With Obinutuzumab or Tazemetostat in Participants With Relapsed/Refractory Follicular Lymphoma and Diffuse Large B-cell Lymphoma
Acronym	
Status	Completed
Study Results	No Results Available
Conditions	Lymphoma
Interventions	Drug: Atezolizumab|Drug: Obinutuzumab|Drug: Tazemetostat
Outcome Measures	Percentage of Participants With Dose Limiting Toxicities (DLTs)|Recommended Phase 2 Dose (RP2D) of Atezolizumab|Obinutuzumab Minimum Serum Concentration (Cmin)|Percentage of Participants With Adverse Events (AEs) Graded According to the National Cancer Institute (NCI) Common Terminology Criteria for Adverse Events version 4.0 (CTCAE v4.0)...
Sponsor/Collaborators	Hoffmann-La Roche
Gender	All
Age	18 Years and Older (Adult, Older Adult)
Phases	Phase 1
Enrollment	96
Funded By	Industry
Study Type	Interventional
Study Designs	Allocation: Non-Randomized|Intervention Model: Parallel Assignment|Masking: None (Open Label)|Primary Purpose: Treatment
Other IDs	GO29383|2014-001812-21
Start Date	18 December 2014
Primary Completion Date	21 January 2020
Completion Date	21 January 2020
First Posted	20 August 2014
Results First Posted	
Last Update Posted	27 January 2020
Locations	City of Hope National Medical Center, Duarte, California, United States|Fort Wayne Neurological Center, Fort Wayne, Indiana, United States|Hackensack University Medical Center, Hackensack, New Jersey, United States…
Study Documents	
URL	https://ClinicalTrials.gov/show/NCT02220842 (accessed on 25 July 2023)

Note: column ‘Outcome Measures’ and ‘Locations’ shortened due to space constraints.

**Table 2:** Features ranked by importance based on Gini Gain.

Feature Name	Explanation
Enrollment	Number of trial participants
Industry-led	Trial led by the industry (true/false)
Location Count	Number of trial locations
Measures Count	Number of outcome measures
Condition Count	Number of medical conditions
Intervention Count	Number of interventions
NCI Sponsorship	Sponsorship includes NCI (true/false)
AES Outcome Measure	Outcome measure includes adverse events (true/false)
Open Masking Label	Trial uses open masking label (true/false)
Biological Intervention	Intervention type includes biological (true/false)
Efficacy Keywords	Title includes efficacy-related keywords (true/false)
Random Allocation	Patient allocation is random (true/false)
US-led	Trial primarily in the US (true/false)
Procedure Intervention	Intervention type includes procedure (true/false)
Overall Survival Outcome Measure	Outcome measure includes overall survival rate (true/false)
Drug Intervention	Intervention type includes drugs (true/false)
MTD Outcome Measure	Outcome measure includes maximally tolerated dose (true/false)
US-included	Trial location includes the US (true/false)
DOR Outcome Measure	Outcome measure includes duration of response (true/false)
Prevention Purpose	Primary purpose is prevention (true/false)
AES Outcome Measure (Lead)	Leading outcome measure is adverse events (true/false)
DLT Outcome Measure	Outcome measure includes dose-limiting toxicity (true/false)
Treatment Purpose	Primary purpose is treatment (true/false)
DLT Outcome Measure (Lead)	Leading outcome measure is dose-limiting toxicity (true/false)
MTD Outcome Measure (Lead)	Leading outcome measure is maximally tolerated dose (true/false)
Radiation Intervention	Intervention type includes radiation (true/false)
Tmax Outcome Measure	Outcome measure includes time of Cmax (true/false)
Cmax Outcome Measure	Outcome measure includes maximum measured concentration (true/false)
Non-Open Masking Label	Trial use non-open masking label (true/false)
Crossover Assignment	Patient assignment is crossover (true/false)

**Table 3:** Key characteristics of training/cross-validation and testing datasets for lymphoma clinical trials.

Characteristics	Training/Cross-Validation Sets (*n* = 871)	Testing Set (*n* = 218)
Percentage of Trials Exceeding 5-Year Completion Time (Target)	40%	40%
Mean Trial Participant Enrollment	49	50
Percentage of Industry-led Trials	46%	48%
Average Number of Trial Locations	6	6
Average Outcome Measures Count	6	6
Average Medical Conditions Addressed	4	4
Average Interventions per Trial	3	2
Percentage of NCI-Sponsored Trials	23%	24%
Percentage of Trials with AES Outcome Measure	34%	34%
Percentage of Trials with Open-Label Masking	91%	92%
Percentage of Titles Suggesting Efficacy	50%	51%
Percentage of Trials Involving Biological Interventions	23%	20%
Percentage of Randomly Allocated Patient Trials	24%	27%

**Table 4:** Performance metrics of machine learning classifiers using 5-Fold cross-validation.

Models/Classifier	Accuracy	ROC-AUC	Precision	Recall	F1-Score
XGBoost (XGB)	0.7442 ± 0.0384	0.7854 ± 0.0389	0.7009 ± 0.0439	0.6286 ± 0.0828	0.6614 ± 0.0633
Random Forest (RF)	0.7371 ± 0.0389	0.7755 ± 0.0418	0.6877 ± 0.0403	0.6286 ± 0.0969	0.6544 ± 0.0667
Logistic Regression (LR)	0.7118 ± 0.0324	0.7760 ± 0.0282	0.6525 ± 0.0487	0.6171 ± 0.0506	0.6323 ± 0.0367
Linear Discriminant Analysis (LDA)	0.7072 ± 0.0393	0.7567 ± 0.0365	0.6457 ± 0.0545	0.6114 ± 0.0388	0.6272 ± 0.0412
Multi-Layer Perceptron (MLP)	0.6717 ± 0.0302	0.7071 ± 0.0593	0.6133 ± 0.0423	0.4914 ± 0.0984	0.5414 ± 0.0684
Gaussian Naïve Bayes (Gaussian NB)	0.5293 ± 0.0169	0.6980 ± 0.0274	0.4571 ± 0.0096	0.9086 ± 0.0194	0.6081 ± 0.0097
K-Nearest Neighbors (KNN)	0.6223 ± 0.0475	0.6487 ± 0.0445	0.5385 ± 0.0762	0.4286 ± 0.0619	0.4786 ± 0.0661
Decision Tree (DT)	0.6464 ± 0.0252	0.6363 ± 0.0317	0.5567 ± 0.0295	0.5771 ± 0.0780	0.5651 ± 0.0502

**Table 5:** Comparative performance of top 4 models on lymphoma testing data.

Model/Classifier	Accuracy	ROC-AUC	Precision	Recall	F1-Score
Random Forest (RF)	0.7248	0.7677	0.675	0.6136	0.6429
Linear Discriminative Analysis (LDA)	0.6927	0.7319	0.6180	0.6250	0.6215
XGBoost (XGB)	0.6881	0.7574	0.6282	0.5568	0.5904
Logistic Regression (LR)	0.6422	0.7281	0.5581	0.5455	0.5517

**Table 6:** Chi-square test—Random Forest vs. other models on correctly predicted trials (testing set).

Total Lymphoma Trials on Testing Set	Correctly Predicted Trials Using Random Forest (RF)	Chi-Square Test Results	
218	158	Correctly Predicted Trials using Linear Discriminative Analysis (LDA)	151
		*p*-Value (RF vs. LDA)	1.45 × 10^−17^
		Correctly Predicted Trials using XGBoost (XGB)	150
		*p*-Value (RF vs. XGB)	4.35 × 10^−30^
		Correctly Predicted Trials using Logistic Regression (LR)	140
		*p*-Value (RF vs. LR)	2.77 × 10^−21^

**Table 7:** Parameter tuning results for the optimal random forest model.

Model/Classifier	Optimal Parameters
Random Forest (RF)	max depth: 20; min sample split: 10; number of trees: 100; bootstrap: false; and seed: 42

**Table 8:** Probability quantile groups with corresponding average duration and 95% confidence intervals.

Probability Quantile Group	Probability Range	Average Duration	Lower Bound (95% CI)	Upper Bound (95% CI)
Q1	0 to 0.1624	1140 days	935 days	1345 days
Q2	0.1624 to 0.3039	1541 days	1235 days	1847 days
Q3	0.3039 to 0.4697	1799 days	1557 days	2041 days
Q4	0.4697 to 0.6291	2150 days	1730 days	2569 days
Q5	0.6291 to 1	2352 days	2005 days	2699 days

## Data Availability

Publicly available datasets were analyzed in this study. These data can be found at https://clinicaltrials.gov (accessed on 25 July 2023).
